# Acute social defeat stress activated neurons project to the claustrum and basolateral amygdala

**DOI:** 10.1186/s13041-022-00987-8

**Published:** 2022-12-20

**Authors:** Masato Tanuma, Misaki Niu, Jin Ohkubo, Hiroki Ueno, Yuka Nakai, Yoshihisa Yokoyama, Kaoru Seiriki, Hitoshi Hashimoto, Atsushi Kasai

**Affiliations:** 1 Laboratory of Molecular Neuropharmacology, Graduate School of Pharmaceutical Sciences, Osaka University, Suita, Osaka 565-0871 Japan; 2 Institute for Open and Transdisciplinary Research Initiatives, Osaka University, Suita, Osaka 565-0871 Japan; 3 Molecular Research Center for Children’s Mental Development, United Graduate School of Child Development, Osaka University, Kanazawa University, Hamamatsu University School of Medicine, Chiba University and University of Fukui, Suita, Osaka 565-0871 Japan; 4 Institute for Datability Science, Osaka University, Suita, Osaka 565-0871 Japan; 5 Department of Molecular Pharmaceutical Sciences, Graduate School of Medicine, Osaka University, Suita, Osaka 565-0871 Japan

**Keywords:** Claustrum, Basolateral amygdala, Activity-dependent labeling, Retrograde tracing, Collateral projections, Stress

## Abstract

We recently reported that a neuronal population in the claustrum (CLA) identified under exposure to psychological stressors plays a key role in stress response processing. Upon stress exposure, the main inputs to the CLA come from the basolateral amygdala (BLA); however, the upstream brain regions that potentially regulate both the CLA and BLA during stressful experiences remain unclear. Here by combining activity-dependent viral retrograde labeling with whole brain imaging, we analyzed neurons projecting to the CLA and BLA activated by exposure to social defeat stress. The labeled CLA projecting neurons were mostly ipsilateral, excluding the prefrontal cortices, which had a distinctly labeled population in the contralateral hemisphere. Similarly, the labeled BLA projecting neurons were predominantly ipsilateral, aside from the BLA in the opposite hemisphere, which also had a notably labeled population. Moreover, we found co-labeled double-projecting single neurons in multiple brain regions such as the ipsilateral ectorhinal/perirhinal cortex, entorhinal cortex, and the contralateral BLA. These results suggest that CLA and BLA receive inputs from neuron collaterals in various brain regions during stress, which may regulate the CLA and BLA forming in a stress response circuitry.

## Introduction

The claustrum (CLA) is a thin sheet like structure located between the insular cortex and the putamen, characterized by its connection with multiple cortical and subcortical brain areas [1–4]. Due to its diverse input–output organization, the CLA has been implicated in functions such as attention and salience [3, 5–8]. Advances in genetic labeling and manipulation techniques have provided detailed insights into the actual function of the CLA, including how the CLA regulates cortical slow wave activity [2, 9]. Recently, our group applied immediate early gene-dependent genetic tools to demonstrate that a subpopulation of CLA neurons activated by acute social defeat stress controls stress-induced anxiety responses [10]. We also observed that most inputs to the CLA activated upon stressor exposure come from the anterior part of the basolateral amygdala (BLA), a brain region critically involved in the processing of emotionally relevant information. However, as the CLA has extensive connections with other brain areas, the CLA is considered part of a stress neurocircuitry; nevertheless, upstream regions that potentially regulate the CLA and other regions of a stress circuitry, such as the BLA, remain unclear.

Axonal branching to form collaterals allows coordinated temporal inputs to their target regions, thus increasing the connectivity of individual neurons and potentially modulating brain states [11–16]. For example, double-projecting neurons to the prefrontal cortex and amygdala in the ventral Cornu Ammonis (CA) 1 region of the hippocampus (HIP) could induce monosynaptic excitatory responses in its target regions, potentially inducing synchronized neuronal activity [17]. These double-projecting neurons also showed preferential activation during contextual fear memory recall in a fear conditioning paradigm, suggesting that collaterals allow the brain to induce a signature activity across brain regions in response to a stimulus. Since the stress responsive CLA neurons and anterior BLA neurons have similar input profiles from cortical and subcortical regions [10, 18, 19], we hypothesized that collaterals to the CLA and BLA may modulate their response to a stressor.

Here we utilized Targeted Recombination in Active Populations (TRAP2) mice [20] for activity-dependent genetic labeling and viral retrograde tracing [21] to detect neurons activated by acute social defeat stress, projecting to both the CLA and BLA. We found brain regions in both the ipsilateral and contralateral hemisphere labeled for neurons projecting to the CLA and BLA. Furthermore, we identified single neurons co-labeled as projecting to the CLA and BLA in the ipsilateral ectorhinal (ECT)/perirhinal cortex (PERI), entorhinal cortex (ENT), and the contralateral BLA. These results suggest that CLA and BLA receive inputs from the collaterals of double-projecting neurons in these brain areas, which may in turn modulate CLA and BLA activity as part of a stress response circuitry.

## Methods

### Animals

A total of three male TRAP2 mice between 7 to 12 weeks of age were used for the experiments. Mice were maintained in group housing (4‒6 mice per group) prior to viral injections and single housed after surgery. Mice were kept on a 12-h light–dark cycle (lights turning on at 08:00 a.m.) in a controlled room temperature, with water and food (CMF, Oriental Yeast, Osaka Japan) available ad libitum.

### Stereotaxic surgery

Mice were anesthetized using 2.0% isoflurane (FUJIFILM Wako Pure Chemical Corp, Osaka, Japan) and head-fixed in a stereotaxic apparatus (David Kopf Instruments, Tujunga, CA). The following stereotaxic coordinates relative to bregma were used for the CLA and BLA, determined using the Franklin and Paxinos mouse brain atlas; CLA (+ 1.60 mm anterior, + 2.25 mm lateral, and − 3.30 mm ventral) and BLA (− 1.00 mm anterior, + 3.10 mm lateral, and − 4.80 mm ventral). Microinjections were performed using a Gastight Syringe (Hamilton, Reno, NV) with a 33-gauge needle attached to an UltraMicroPumpIII with a Micro4 controller (World Precision Instruments, Sarasota, FL). Adeno-associated virus (AAV) solutions were injected at a rate of 50 nL/min. The needle was subsequently left in place for 10 min to allow for even spread of the solution, before being slowly removed at 1 mm/min to prevent backflow. All mice received an intraperitoneal injection of buprenorphine (0.1 mg/kg; Otsuka Pharma, Tokyo, Japan) and gentamicin (10 mg/kg, Sigma-Aldrich, St Louis, MO) after surgery to assist on recovery.

### Social defeat stress

The CD-1 mice used as aggressors were prescreened for aggressive behaviors using previously described parameters [22]. Test mice undergoing the social defeat stress were placed into the aggressor CD-1 mouse home cage for 10 min before being returned to their home cage.

### Activity-dependent retrograde labeling

Activity-dependent genetic labeling of TRAP2 mice with retrograde AAVs was performed as previously described [10]. Briefly, a single brain hemisphere in TRAP2 mice was unilaterally injected with 100 nL of AAVrg-hSyn-DIO-EGFP (≥ 7 × 10^12^ particles/mL; gifted from Bryan Roth; Addgene viral prep # 50,457-AAVrg; http://n2t.net/addgene:50459; RRID:Addgene_50457) in the CLA and 100 nL of AAVrg-hSyn-DIO-mCherry (≥ 7 × 10^12^ particles/mL; gifted from Bryan Roth; Addgene viral prep # 50,459-AAVrg; http://n2t.net/addgene:50459; RRID:Addgene_50459) in the BLA. After one week of post-surgery recovery, single housed mice received an injection of 2 mg tamoxifen (Cayman Chemical, Ann Arbor, MI) dissolved in a 1:9 (v/v) mixture of ethanol (FUJIFILM Wako Pure Chemical) and corn oil (Sigma-Aldrich) at 20 mg/mL. Mice were exposed to a single bout of social defeat stress 6 h after the tamoxifen injection, and subsequently single housed in a serene environment for the next 48 h.

### Whole brain imaging

Whole brain imaging was performed using the block-face serial microscopy tomography (FAST) as previously described [23, 24]. Briefly, brains transcardially perfused with 4% paraformaldehyde (Nacalai Tesque, Kyoto, Japan) were embedded in a 4% agarose gel (Nacalai Tesque) dissolved in phosphate-buffered saline. Serial brain section images were acquired as a mosaic of field-of-views, with 20% overlap for each image in the x–y plane and 30 µm overlap across adjacent sections in the z direction. The resulting pixel size was 1.0 µm in the x–y plane and 5.0 µm in the z direction. Acquired mosaics were reconstructed using FASTitcher with the suggested parameters [24]. The cell numbers for each brain region were manually counted using the Cell Counter plugin on ImageJ.

### Schematics of anatomical structures

All schematics of brain structures were created using the Franklin and Paxinos mouse brain atlas [25].

### Abbreviations for brain areas

The abbreviations for the designated brain areas used in this manuscript are in accordance with those provided in the Allen Brain Reference Atlas (http://atlas.brain-map.org). Please refer to the list of abbreviations below.

## Results

**Fig. 1:**
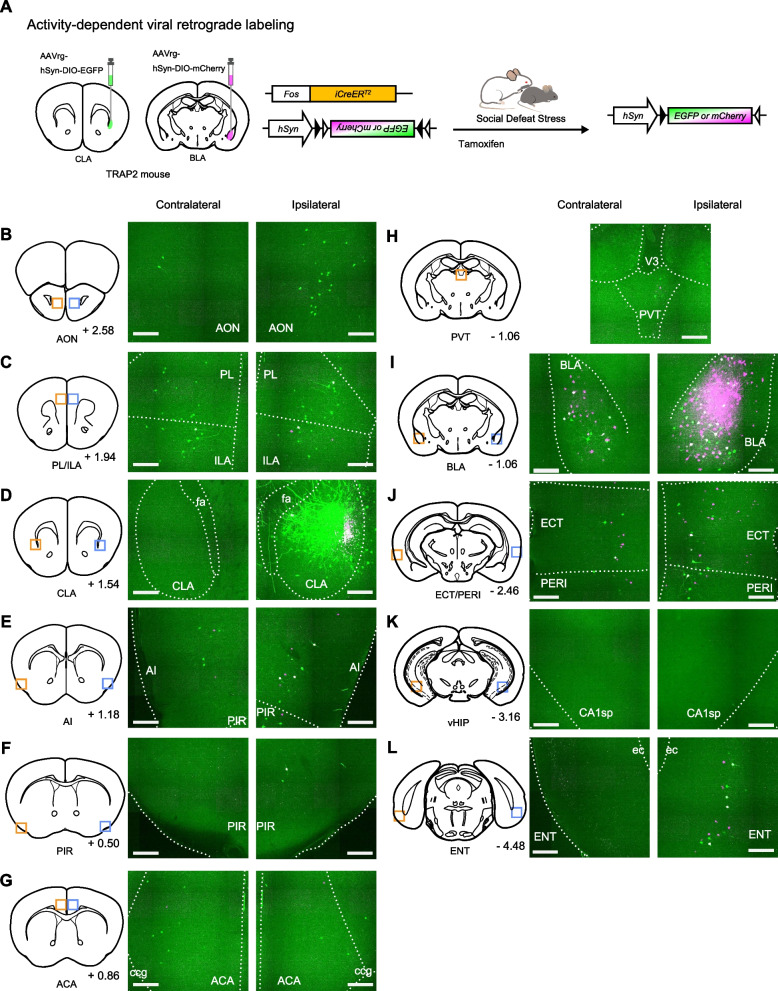
Stress activated neurons projecting to the CLA and BLA. **A** Schematic for Cre-dependent viral retrograde labeling in the CLA and BLA. **B**–**L** Representative images from the brain regions analyzed in both hemispheres. Left, contralateral hemisphere; right, ipsilateral hemisphere. The single image from the PVT includes both hemispheres. All images correspond to one representative mouse. Retrogradely labeled neurons projecting to the CLA (green) and BLA (magenta). Orange boxes in the coronal diagram show the magnified area on the contralateral side, blue boxes show the magnified area on the ipsilateral side. Numbers to the bottom right of each coronal diagram show the distance (mm) from bregma on the anterior–posterior axis. Coronal diagrams modified from the Paxinos and Franklin’s The Mouse Brain. Scale bars, 0.2 mm

To identify the neurons activated by social defeat stress projecting to the CLA and BLA, we performed Cre-dependent retrograde tracing as previously described [10]. We unilaterally microinjected two retrograde AAV vectors, one carrying EGFP (hSyn-DIO-EGFP) into the anterior part of the CLA and another carrying mCherry (hSyn-DIO-mCherry) into the anterior part of the BLA of the same hemisphere of TRAP2 mice (Fig. 1A). The anterior part of the CLA was targeted since the anterior part of the CLA shows the highest increase in cells activated by psychological stress along the rostocaudal axis [10]. We specifically targeted the anterior part of the BLA as the distribution of stress activated BLA neurons projecting to the CLA is abundant there [10]. Mice were then subjected to a single bout of social defeat stress 6 h after an intraperitoneal tamoxifen injection to specifically tag neurons activated by exposure to the stressor.

**Fig. 2:**
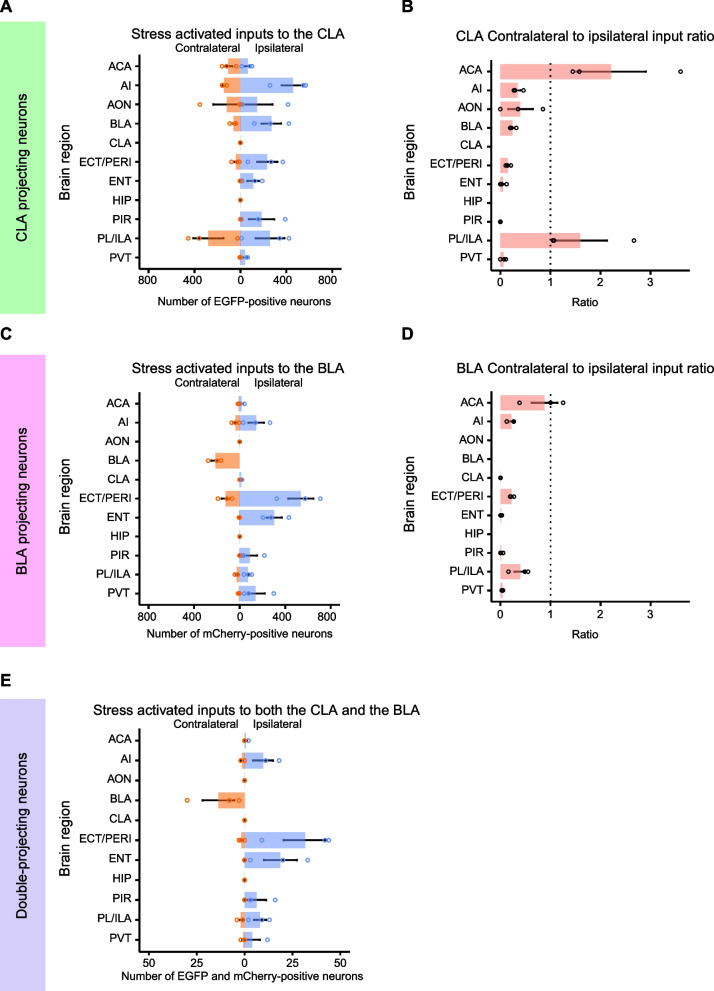
Quantification of stress activated neurons projecting to the CLA and BLA. **A** Number of EGFP-positive neurons in each brain region per hemisphere (contralateral, orange; ipsilateral, blue). Neurons in the ipsilateral CLA were not quantified as it was the AAV injection site. **B** Ratio of contralateral to ipsilateral inputs to the CLA in each brain region analyzed. The CLA ratio was not calculated. **C** Number of mCherry-positive neurons in each brain region, counted by hemisphere (contralateral, orange; ipsilateral, blue). Neurons in the ipsilateral BLA were not quantified as it was the AAV injection site. **D** Ratio of contralateral to ipsilateral inputs to the BLA in each brain region analyzed. The BLA ratio was not calculated. **E** Number of EGFP-positive and mCherry-positive double-projecting neurons in each brain region per hemisphere (contralateral, orange; ipsilateral, blue). Neurons in the ipsilateral CLA and BLA were not quantified as they were AAV injection sites. All data are presented as the mean ± s.e.m

We first analyzed the EGFP-positive neurons projecting to the CLA. We focused our analysis on brain regions that we previously reported to have a rich population of retrogradely labeled neurons from the CLA, and some additional areas. Multiple brain regions expressing EGFP were observed across the brain, from cortical regions including the agranular insular (AI), anterior olfactory (AON), ENT, ECT/PERI, piriform (PIR), and the prelimbic (PL)/infralimbic (ILA) cortices, to subcortical regions including the BLA and the paraventricular nucleus of the thalamus (PVT) (Fig. 1B–L). Although previous reports showed monosynaptic inputs from the ventral HIP to the CLA [1, 2], we did not observe any EGFP-positive neurons in the HIP across animals analyzed (Fig. 1I). Neither did we observe EGFP-positive neurons in the CLA of the contralateral hemisphere (Fig. 1C). We quantified the number of labeled neurons in each brain region by hemisphere, as recent findings suggest that even if the CLA predominantly receives ipsilateral inputs, specific brain areas such as the prefrontal cortices also provide contralateral inputs [1, 4]. Analyses of ipsilateral inputs to the CLA showed a similar pattern to our previous work [10], with a large number of EGFP-positive neurons being found in the AI, BLA, PL/ILA, ECT/PERI, PIR, and ENT (Fig. 2A). As expected, the analysis of contralateral inputs to the CLA revealed a different pattern from ipsilateral inputs. The PL and ILA provided most contralateral inputs to the CLA, while only a small number of contralateral inputs were observed in the ECT/PERI and ENT (Fig. 2A). The ratio of contralateral to ipsilateral inputs revealed that while most inputs to the CLA were preferentially ipsilateral (average ratio < 1), the anterior cingulate cortex (ACA) and PL/ILA inputs were conversely enriched in the contralateral hemisphere (Fig. 2B), consistent with previous monosynaptic retrograde tracing in the CLA [1, 3, 4]. These results show that the CLA receives brain wide stress related inputs; with abundant ipsilateral inputs from the AI, BLA, PL/ILA, ECT/PERI and ENT; and contralateral inputs from the PL/ILA and ACA.

Next, we analyzed the mCherry-positive neurons projecting to the BLA. Similar to inputs to the CLA, we observed mCherry expression in cortical and subcortical brain regions, including the ECT/PERI, ENT, AI, and the PVT (Fig. 1B–L). mCherry-positive neurons were not observed in the HIP, despite the presence of HIP inputs to the BLA (Fig. 1I) [19]. Although we observed a large number of EGFP-positive BLA neurons projecting to the CLA, we observed very few mCherry-positive neurons in the CLA (Fig. 2C). Quantification of the number of labeled neurons by hemisphere revealed that the BLA receives many ipsilateral inputs from the ECT/PERI and ENT (Fig. 2C), whereas inputs from the contralateral hemisphere predominantly arose from the BLA and ECT/PERI (Fig. 2C). Similar to the CLA, contralateral projections from the ENT were minimal. The ratio of contralateral to ipsilateral inputs to the BLA showed that inputs from all the brain regions analyzed were preferentially ipsilateral, even if the ACA and PL/ILA were the top two brain regions in terms of the contralateral to ipsilateral ratio, albeit with a small number of neurons (Fig. 2D). These results show that the BLA receives diverse stress related inputs, mainly from ipsilateral cortical areas as well as a characteristic interhemispheric input from the contralateral BLA.

**Fig. 3:**
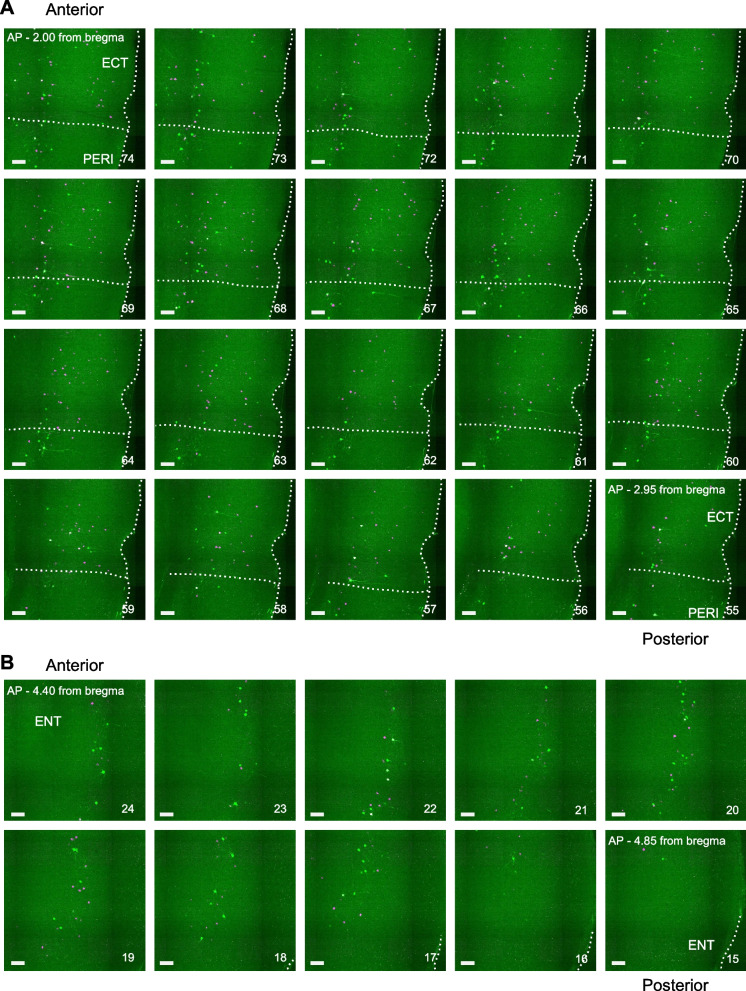
Serial images of stress activated neurons in the ipsilateral ECT/PERI and ENT. Serial magnified images of the ipsilateral ECT/PERI spanning − 2.00 to − 2.95 mm from bregma (**A**), and ipsilateral ENT spanning − 4.40 to − 4.85 mm (**B**) at 0.05 mm intervals. Scale bars, 0.2 mm. Numbers to the bottom right of each serial image show the slice number from the whole brain imaging experiment, ranging from 74 (anterior) to 55 (posterior) (**A**), and 24 (anterior) to 15 (posterior) (**B**)

**Fig. 4:**
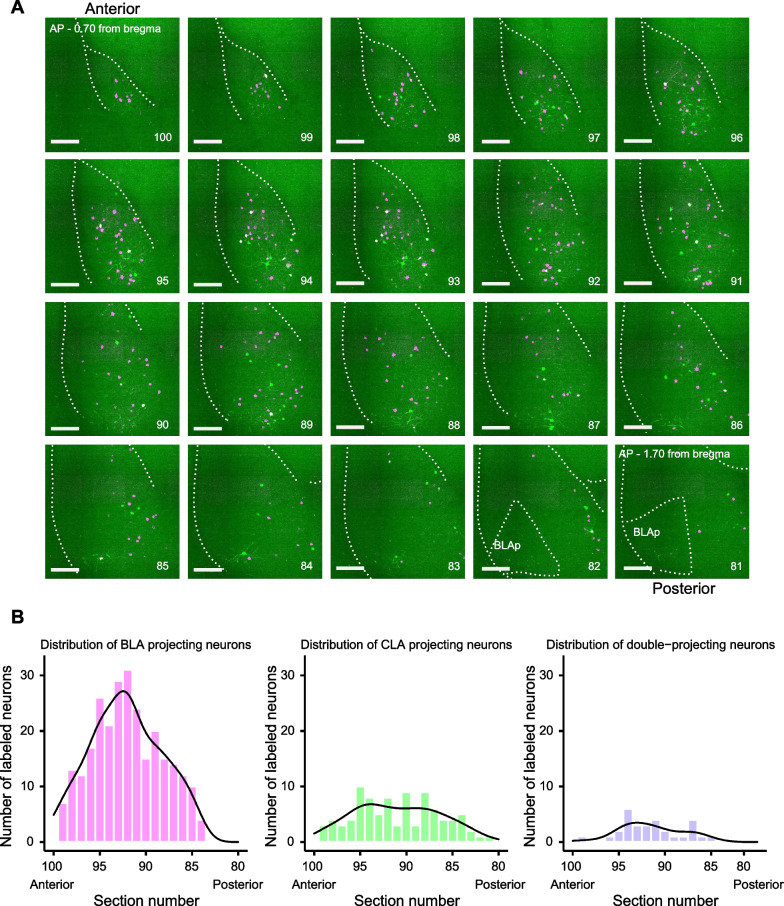
Distribution of stress activated neurons in the contralateral anterior BLA. **A** Serial magnified images of the contralateral anterior BLA spanning − 0.70 to − 1.70 mm from bregma at 0.05 mm intervals. Numbers to the bottom right of each serial image show the slice number from the whole brain imaging experiment, ranging from 100 (anterior) to 81 (posterior). Scale bars, 0.2 mm. BLAp, posterior part of the BLA. **B** Histograms of BLA projecting (magenta), CLA projecting (green), and CLA and BLA double-projecting (purple) neurons across the serial images shown in A. Black lines show the kernel density estimation for each histogram. The peak for each density plot is found between serial sections 95 and 90

These results indicate that both the CLA and BLA receive inputs from numerous cortical and subcortical brain regions activated by social defeat stress, with numerous shared brain regions. We next investigated whether these brain regions both retrogradely labeled from the CLA and BLA have double-projecting single neurons providing inputs to both brain regions, which could potentially regulate stress related information. To this end, we analyzed and quantified the number of CLA projecting EGFP and BLA projecting mCherry double-positive neurons in each brain region analyzed independently in the ipsilateral and contralateral hemispheres (Fig. 2E). Ipsilateral double-positive neurons were observed in multiple brain regions such as the ECT/PERI, ENT, AI, PL/ILA and PIR. (Fig. 2E). The ECT/PERI and ENT regions showed the largest number of double-positive neurons in the ipsilateral hemisphere, accounting for most such inputs (Fig. 3). On the contralateral hemisphere, there were few double-positive neurons, except for the contralateral BLA, where a distinct population was observed. Because we previously identified the anterior BLA as providing the largest fraction of stress-responsive inputs to the CLA, we subsequently analyzed the distribution of double-projecting contralateral BLA neurons (Fig. 4A). We find the double-projecting contralateral BLA neurons were also distributed towards the anterior BLA (− 0.70 to − 1.70 mm from bregma) than the posterior BLA (− 1.60 to − 2.70 mm from bregma) (Fig. 4B). These results show that double-projecting neurons to both the CLA and BLA and activated by social defeat stress are found across the brain mostly in ipsilateral cortical structures and the contralateral BLA.

## Discussion

In this report, we investigated the neurons activated by acute social defeat stress projecting to the CLA and BLA using a combination of activity-dependent labeling and viral retrograde tracing. We found that the CLA and BLA receive inputs from overlapping brain regions. Ipsilateral projections from the AI, BLA, PL/ILA, ECT/PERI, and ENT, and contralateral projections from the PL/ILA and ACA to the CLA were abundant. The BLA also received ipsilateral projections from the ECT/PERI, ENT, and AI, while contralateral inputs mainly came from the contralateral BLA. Moreover, single double-projecting neurons were also found across multiple brain areas. The ECT/PERI and ENT showed the most co-labeled neurons on the ipsilateral side while the BLA had the most on the contralateral side. These results suggest the CLA and BLA have multiple upstream common areas; double-projecting neurons in those regions may coordinate their neuronal activity in response to a psychological stressor.

The observation of retrogradely labeled double-positive neurons suggests the possibility of collateralization in those projection neurons. Collateralization is a notable characteristic of projection neurons that allows for coordinated control of multiple brain regions by a single neuron [11, 16]. The presence of neurons forming collaterals onto the CLA and BLA in multiple brain regions such as the ENT, ECT/PERI, PL/ILA, AI, and the contralateral BLA, all areas associated with the processing of emotionally relevant information, suggest that CLA and BLA activity may be coordinated by these upstream regions forming a stress neurocircuitry. Temporally synchronized inputs may produce a signature activity that essentially modulates brain states, as suggested by others [11, 16], supporting our previous finding that the CLA and BLA were the top contributors to discriminating stressed brains from non-stressed brains. [10].

An interesting finding was that the contralateral BLA was also rich in double-projecting neurons to the CLA and BLA of the opposite hemisphere. The anatomical interhemispheric connections of the BLA have previously been documented in rodents [26]; a recent publication reported that this contralateral innervation potentiates the synaptic responses of both BLA populations, thereby increasing synchronized activity [27]. Contralateral projecting BLA neurons have collaterals to other brain areas [27, 28]; here we provide evidence of collaterals to the contralateral CLA. Additionally, collaterals are capable of activating direct and indirect pathways to a particular brain area, as shown by the fact that double-projecting ventral CA1 neurons activated BLA neurons and PL/ILA neurons projecting to the BLA in a ventral CA1-BLA-PL/ILA circuit [17]. Taken together, the double-projecting contralateral BLA neurons may similarly activate downstream signals in the CLA both directly and indirectly via potentiation of BLA neurons projecting to the CLA. Such a mechanism may allow the efficient transfer of stress related information, but further research is needed to clarity this point.

The distribution of contralateral BLA projecting stress activated BLA neurons increased towards the anterior BLA (− 0.70 to − 1.70 mm from bregma; Fig. 4), which contradicts a previous report stating that contralateral projecting BLA neurons were concentrated at the posterior part of the BLA (− 1.60 mm from bregma) [27]. This discrepancy can be explained as either functional differences in stress response between the anterior and posterior parts of the BLA or differences in experimental conditions, notably the site of virus infection along the anterior/posterior axis.

Previous anatomical studies have concluded that the CLA has strong reciprocal connections with frontal cortical structures, and a few subcortical regions such as the BLA [1]. As the retrograde tracing in this report and our previous work [10] show that BLA neurons projecting to the CLA are activated by social defeat stress, we expected CLA neurons projecting to the BLA to be activated by the same stimulus. However, only a few CLA neurons projecting to the BLA were activated by social defeat stress, suggesting a unidirectional information flow from the BLA to the CLA. Conversely, we observed activated inputs from the PL/ILA to the CLA, suggesting feedback from the frontal cortices. This enhances the recent view that the CLA functions as a limbic-motor interface, transferring and possibly processing emotional information from subcortical limbic areas, generating preparatory activity, and eliciting appropriate movement and behaviors via communication with cortical areas [29–31].

### Limitations

Despite showing the existence of potential collateral inputs to both the CLA and BLA, the experiments in this report used an AAV capsid developed and targeted for retrograde transport from axon terminals [21]. Hence, the neurons retrogradely labeled do not necessarily possess monosynaptic connections with the CLA and BLA. Further experiments using other methods such as rabies virus injection are needed to confirm monosynaptic connections, even if the retrogradely labeled brain regions and their ipsilateral/contralateral profiles were in agreement with previous monosynaptic research. Because the CLA is very thin and difficult to exclusively target, there may be potential for leakage of AAVs into adjacent brain regions, such as the AI. Additionally, factors such as leaky expression of Cre recombinase and infection rates of AAV particles in the retrogradely labeled regions should also be considered, and thus validation with immunohistochemistry and by calculating the ratio between Cre-dependently/Cre-independently labeled neurons may be necessary. Also, we cannot entirely rule out the possibility of neurons being labeled through other stimuli such as exposure to a novel environment during activity-dependent labeling, since the CLA can be activated by placement in a novel environment [32]. Accordingly, these limitations may need to be taken into consideration when interpreting the present results.

## Data Availability

All data used in this paper are available upon reasonable request to the corresponding author.

## Abbreviations

AI: Agranular insular
AAV: Adeno-associated virus
ACA: Anterior cingulate area
AON: Anterior olfactory nucleus
BLA: Basolateral amygdala
CA: Cornu Ammonis
CLA: Claustrum
ECT: Ectorhinal cortex
ENT: Entorhinal cortex
ILA: Infralimbic area
HIP: Hippocampal region
PERI: Perirhinal cortex
PIR: Piriform area
PL: Prelimbic area
PVT: Paraventricular nucleus of the thalamus
TRAP2: Targeted recombination in active populations
